# Design and evaluation of a treatment programme for Spanish adolescents with overweight and obesity. The EVASYON Study

**DOI:** 10.1186/1471-2458-9-414

**Published:** 2009-11-15

**Authors:** David Martinez-Gomez, Sonia Gomez-Martinez, M Angeles Puertollano, Esther Nova, Julia Wärnberg, Oscar L Veiga, Amelia Martí, Cristina Campoy, Jesus M Garagorri, Cristina Azcona, M Pilar Vaquero, Carlos Redondo-Figuero, Manuel Delgado, J Alfredo Martínez, Miguel Garcia-Fuentes, Luis A Moreno, Ascension Marcos, EVASYON Study Group

**Affiliations:** 1 Department of Metabolism and Nutrition, Instituto del Frío, Institute of Food Science and Technology and Nutrition (ICTAN), Spanish National Research Council (CSIC), Madrid, Spain; 2 Department of Physical Education, Sport and Human Movement, University Autonomous of Madrid, Madrid, Spain; 3 Department of Physiology and Nutrition, University of Navarra, Pamplona, Spain; 4 Department of Pediatrics, University of Granada, Granada, Spain; 5 School of Health Sciences, University of Zaragoza, Zaragoza, Spain; 6 Department of Paediatrics, Clinica Universitaria, Pamplona, Spain; 7 Department of Pediatrics, University of Cantrabria, Cantrabria, Spain; 8 Department of Physical Education, University of Granada, Granada, Spain

## Abstract

**Background:**

The prevalence of overweight and obesity (OW/OB) among adolescents worldwide has increased since the 60 s. Spain has reached one of the highest OW/OB prevalence rates among adolescents from European countries. The aim of this methodological paper is to describe the design and evaluation in the EVASYON study (Development, implementation and evaluation of the efficacy of a therapeutic programme for adolescents with OW/OB: integral education on nutrition and physical activity).

**Methods/Design:**

The EVASYON was planned by a multidisciplinary team to treat OW/OB in Spanish adolescents. The EVASYON is a multi-centre study conducted in 5 hospitals in 5 Spanish cities (Granada, Madrid, Pamplona, Santander and Zaragoza) and two hundred and four OW/OB Spanish adolescents were recruited for this intervention. The treatment was implemented for approximately one-year follow-up. The adolescents were treated in groups of a maximum of 10 subjects; each group had 20 visits during the treatment period in two phases: intensive during the first 2 months (1^st ^to 9^th ^visits), and extensive during the last 11 months (10^th ^to 20^th ^visits). In order to assess the efficacy of the treatment, 8 dimensions were measured: diet; physical activity and fitness; eating behaviour; body composition; haematological profile; metabolic profile; minerals and vitamins; immuno-inflammatory markers. Moreover, genetic polymorphisms were also determined.

**Discussion:**

The treatment programme developed in the EVASYON study was designed as a national pilot study to be implemented as an effective treatment for adolescents with OW/OB into the Spanish Health Care Service.

## Background

Adolescence is characterized by important changes in body size and composition. Obesity started to appear as a public health problem in the last decades of the 20th century [1]; currently acquiring epidemic dimensions. Obesity is a multifactorial condition, with many biological, genetic, social and environmental influences affecting its development [2,3]. In most cases, obesity is the result of inappropriate eating behaviour, which is becoming a major dietary, psychological and social problem in developed countries [4]. A great deal of recent literature has focused on inadequate eating and physical activity patterns, especially TV watching and other types of sedentary behaviour [5,6]. However, the development of overweight in adolescence may depend on the prevalence of many other obesigenic risk factors [7], such as ethnicity and socioeconomic status [8-12]. As a result, overweight leads to adverse short-term consequences during childhood, such as psychosocial problems, and long-term results have also been reported during adulthood [13,14].

Overweight prevalence among adolescents around the world has increased since the 1960s. Approximately 14-15% of all 15-yr-old youngsters in the United States may be classified as obese [15]. Similarly, a dramatic increase in the prevalence of overweight and obesity among children and adolescents in the European Union (EU) has occurred in the last 20 years. About 30% of European children are overweight and approximately one quarter of them is obese [16]. Overweight and obesity prevalence rates in Spanish adolescents were similar to those observed in other European countries such as Greece, Portugal, England or Belgium [17,18]. However, obesity prevalence in some countries seems to be levelling-off [19-21]. This could be explained, in some cases, because obesity prevalence was already very high; but, in other cases, it could be due to the national public health efforts showing promising positive results. In spite of these figures and to be the prevention the first public health strategy to fight against obesity, we cannot forget the huge rates of overweight children and adolescents who must be treated.

It is widely accepted that treating childhood overweight is an important contribution to the multilevel response to the obesity epidemic [2]. Effective treatment for most obese children and adolescents remains elusive. Management protocols involving behaviour modifications, family support, and lifestyle changes are difficult to put into practice and may require the input of multi-disciplinary professional teams [22,23]. In fact, lifestyle changes require a high degree of motivation and active participation from the adolescents and their relatives [1]. Weight-loss programmes in children and adolescents have shown a full range of results. Isolated (e.g. physical activity, sedentary behaviours, diet) and combined programmes have been investigated with relevant findings [24-26]. Overall, combined programmes tend to be more effective in children and adolescents because beneficial modifications are kept for long-term. On the other hand, medication or surgical interventions in these ages have been considered as a possibility in extreme cases.

The main aims of the EVASYON study (Development, implementation and evaluation of the efficacy of a therapeutic programme for adolescents with overweight and obesity: integral education on nutrition and physical activity) were: 1) to develop a treatment programme including education on nutrition and physical activity, 2) to implement this programme for one year in Spanish adolescents with overweight and obesity and 3) to evaluate its efficacy.

For comparative and popularization purposes with previous and future studies, the aim of this paper was to describe the design and evaluation methods during the EVASYON study.

## Methods/Design

### Study design and sample size estimation

The design of the EVASYON Study is an interventional study in a cohort of overweight and obese adolescents aged 13 to 16 years. Despite the lack of control group, we aim to assess the determinants of the treatment effectiveness.

In order to estimate the sample size, we considered the results obtained in the AVENA study, a multicenter evaluation of the nutritional status of Spanish adolescents [27], showed that body fat reached the greatest variance among anthropometrical variables [17] and was thus considered as the main variable in the EVASYON study. In order to achieve a 2.7% reduction between the estimated mean and the sampling mean with a statistical power equal to 90% and an alpha risk of 0.05, one hundred and fifty-three participants were necessary, being the rest of the variables correctly described and included for this sampling size. The sampling size was increased by 25% to avoid a reduction of the statistical significance of the results due to possible dropouts. The current study included a nutritional treatment and an educational programme on diet, nutrition concepts and physical activity. Two hundred and four Spanish male and female adolescents aged 13-16 years with overweight and obesity, were the target population to be treated at 5 paediatric units in 5 hospitals from 5 different Spanish cities (Granada, Madrid, Pamplona, Santander and Zaragoza). A pilot study was previously carried out including 6 participants from each hospital. This pilot study allowed us to harmonize nutrition, psychology and physical activity interventional protocols. Moreover, preliminary measurement procedures were tested in this pilot group of adolescents.

**Figure 1:**
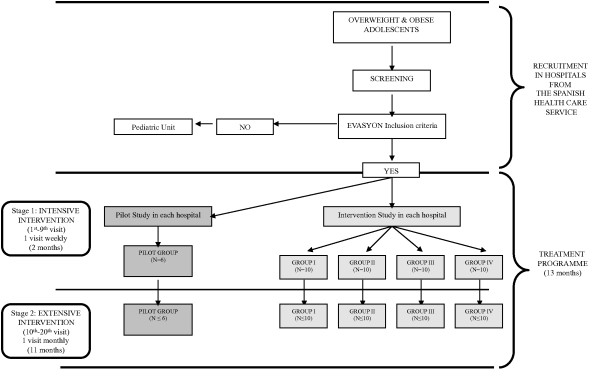
**EVASYON study design in each hospital involved in the treatment programme**.

Before starting the EVASYON programme, a screening was performed of all candidates. Several inclusion criteria were defined to homogenize sample characteristics. Inclusion criteria in the EVASYON programme are shown in Table 1. The EVASYON treatment programme has been conducted in small groups from 9 to 11 patients. Therefore, each hospital involved in the current study had to manage at least 5 intervention groups including the pilot study. The treatment programme was carried out in each group during approximately one year including twenty visits within two specific stages (Figure 1): 1) Intensive intervention (9 visits): participants visited the hospitals weekly for 2 months. Paediatricians explained the patients several motivational strategies, life and time management strategies including physical activity recommendations or sleep time, nutritional advice, family involvement, etc. In this stage, one-week objectives were defined; 2) Extensive intervention (11 visits): participants visited the hospital monthly during 11 months. In this stage, objectives for the adolescents were to be accomplished in one month's time.

**Table 1:** Inclusion and exclusion criteria in the EVASYON study

Inclusion criteria	Exclusion criteria
1. To be 13-16 years aged	1. To consume any pharmacological treatment
2. To be overweight or obese in agreement with Cole's criteria [49]	2. Anorexia, bulimia or other eating disorder diagnosis, except binge eating disorder
3. To be Spanish or to have been educated in Spain	
4. Do not suffer other diagnosed disease	

### Assessment during follow-up

Nine measurement categories were assessed: 1) Diet; 2) Physical activity and health-related physical fitness; 3) Psychological profile; 4) Body composition; 5) Haematological profile; 6) Biochemistry and metabolic profiles; 7) Mineral and vitamin profile; 8) Immunological profile; 9) Genetic profile.

**Figure 2:**
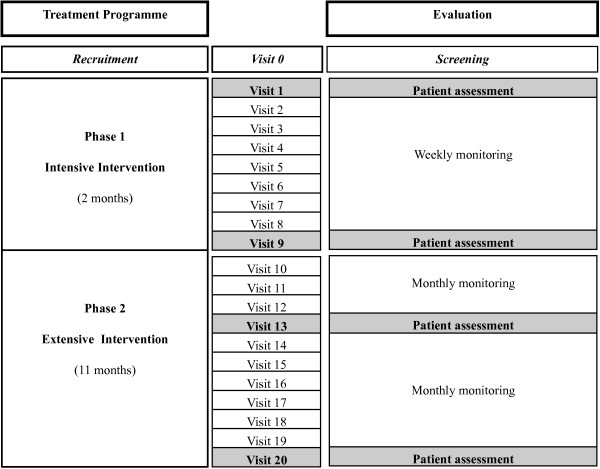
**Evaluation during the EVASYON treatment programme**.

All the parameters of each measurement category, excluding genetic profile were assessed at least at four points (Figure 2): baseline (visit 1), at the end of the intensive intervention (visit 9), at mid point of the extensive intervention (visit 13), and at the end of the EVASYON treatment programme (visit 20).

### Applied Methodology

#### Dietary intake assessment methods

A trained dietician conducted face-to-face interviews with participants and their parents (father, mother or tutor) at the beginning of the programme and visits 1, 9, and 20 (see Figure 2). In visits 1, 9, 13, and 20 interview information about food intake (72-h dietary record), dietary patterns, and nutrition knowledge was collected in order to evaluate fulfilment of the recommended diet as well as changes in food intake habits during the intervention programme.

Moreover, a semi-quantitative food-frequency questionnaire, which was previously validated in Spain [28,29], was completed. It contained 132 food items divided into the following categories: dairy products, meat and eggs, fish, fruit and vegetables, legumes, potatoes and cereals, nuts, oils and fat, sweets and beverages. For each food item, an average portion size was specified, and participants and their parents were asked how often they had consumed that unit throughout the previous period having nine options for the frequency of intake (ranging from never/almost never to at least six times per day).

Data of food intake by 72-h dietary record were transformed into food volume/weight (in mL or g). Nutrient intake scores were computed with and *ad hoc *computer programme specifically developed to this aim. A trained dietician updated the nutrient data bank using the latest available information in food-composition tables from Spain [30,31].

#### Physical activity and health-related physical fitness

Physical activity was assessed applying a combination of methods. Participants in the EVASYON study wore the ActiGraph GT1M activity monitor (ActiGraph™, LLC, Fort Walton Beach, FL, USA). The ActiGraph GT1M is a small and lightweight uniaxial accelerometer (3.8 × 3.7 × 1.8 cm, 27 g) validated widely in laboratory and free-living conditions with children and adolescents [32]. According to previous studies in Spanish adolescents [33], participants wore the monitor for 7 consecutive days and 15-s epochs were used in each measurement [34]. The adapted Spanish version from the Physical Activity Questionnaire for Adolescents (PAQ-A) was also used in the study to assess physical activity [35]. The PAQ-A measured 1 to 5 physical activity levels in each adolescent [36]. Moreover, other questionnaires were administered to assess patterns and determinants related to physical activity: sedentary behaviour, physical activity stages, family influence, change strategies, self-efficacy and environmental factors.

In the EVASYON study, health-related physical fitness was assessed using the validated and standardized tests included in the EUROFIT [37] and FITNESSGRAM [38] test batteries. These methodologies have been used in the AVENA and HELENA studies [39] to assess physical fitness in adolescents between 13 and 16 years of age in Spain and other European countries, respectively. The 5 fitness tests selected for this study were:

##### a) Course navette or 20-m Shuttle run test

The progressive 20-m shuttle-run test published by Leger and Lambert in 1982 and revised in 1988 [40] is one of the most widely used field tests to assess cardiorespiratory fitness among children and adolescents. Subjects run as long as possible back and forth across a 20-m space at a specified music protocol that gets 0.5 km/h faster each minute or period. Last 0.5 period completed is the individual score and VO_2_max may be estimated with the Leger equation.

##### b) Handgrip strength

Subjects perform the test in a standard bipedal position and with the arm in complete extension without touching any part of the body with the hand-dynamometer (TKK 5101^©^; Takei, Tokyo, Japan). Dynamometer was adjusted by sex and hand size for each subject [41]. Handgrip strength test provides some results about the maximal isometric force that can be generated mainly by the hand and arm.

##### c) Standing broad jump

In standing position, subject jumps as far as possible trying to land with both feet together. The score is the distance between the last heel-mark and the take-off line. Standing broad jump assesses lower-limb explosive strength.

##### d) 4 × 10-m shuttle-run

4 × 10-m is an adaptation of the 5 × 10-m shuttle run test included in the EUROFIT battery but maintaining the same characteristics. Velocity, agility and coordination are assessed in this test. Participants run 4 × 10-m (back and forth) as fast as possible.

##### e) Back-saver sit and reach

Proposed in the FITNESSGRAM battery, this test measures the flexibility of the hamstring muscles. The test is performed with a standard and sturdy box with a scale on the top of the box. Back-saver sit and reach is similar to the traditional sit and reach except that the measurement is performed on one side at a time, so each side has its individual score.

#### Psychological and eating behaviour assessment

The questionnaires used during the EVASYON Study were the following:

##### a) AF-5 Self-Concept Questionnaire

In order to assess self-esteem, the AF-5 [42] multidimensional Self-Esteem Scale appraises five personal aspects: academic, social, emotional, family, and physical dimensions.

##### b) Anorectic Behaviour Observation Scale (ABOS)

The ABOS scale was developed to evaluate patient's symptoms based on the relatives' description of the subject's eating behaviour. This test is useful in a clinical setting for evaluation of patients with eating disorders. The original version [43] evaluated three factors: 1) eating behaviour, related to weight and foods, 2) bulimic-like behaviour and 3) hyperactivity.

##### c) Eating Disorder Inventory (EDI-2)

EDI-2 is a self-report instrument that assesses the cognitive and behavioural characteristics commonly found in individuals with eating disorders [44]. It is organized into eleven subscales that contain varying numbers of items. The subscales are as follows: drive for thinness, body dissatisfaction, bulimia, ineffectiveness, perfectionism, interpersonal distrust, interoceptive awareness, maturity fears, ascetic behaviour, impulsive behaviour and social insecurity.

#### Body Composition, pubertal development and resting blood pressure

The anthropometry protocol used in the EVASYON study was identical to the standardized protocol used in the AVENA study with more than 2000 Spanish adolescents [45].

Each measurement was taken three times but not consecutively. A complete set of measurements was performed and then repeated twice more. Weight and height are obtained by standardized procedures. Body mass index is calculated as weigh/height squared (kg/m^2^). Skinfold thickness were measured on the left side of the body [46] to the nearest 0.1 mm with a skinfold caliper (Caliper Holtain; Holtain Ltd., Walles, UK) at the following sites: 1) triceps, halfway between the acromion process and the olecranon process; 2) biceps, at the same level as the triceps skinfold, directly above the centre of the cubital fossa; 3) subscapular, about 20 mm below the tip of the scapula, at an angle of 45° to the lateral side of the body; 4) suprailiac, about 20 mm above the iliac crest and 20 mm towards the medial line; 5) thigh, in the midline of the anterior aspect of the thigh, midway between the inguinal crease and the proximal border of the patella; 6) calf, at the level of maximum calf circumference, on the medial aspect of the calf.

The five circumferences are measured in centimetres with an inelastic tape to the nearest millimetre. In general, for these measurements, the subject is in a standing position. For measuring the arm circumference relaxed, the subject stands relaxed with his/her side to the observer, the arm hanging freely at the side; the tape is passed around the arm at the level of the midpoint of the upper arm. For measuring upper arm circumference flexed (biceps circumference) the subject contracts muscle biceps as much as possible, and the tape is passed around the arm so that it touches the skin surrounding the maximum circumference. To measure the waist circumference, the tape is applied horizontally midway between the lowest rib margin and the iliac crest, at the end of gentle expiration. The hip circumference measurement is taken at the point yielding the maximum circumference over the buttocks, with the tape held in a horizontal plane. Proximal thigh circumference is measured just below the gluteal fold and perpendicular to its long axis; the subject stands erect with the feet slightly apart and the body mass evenly distributed between both legs. In addition to the anthropometry measurements, in Zaragoza and Granada, we used laboratory techniques. Participants in these cities (n≈80) were assessed by bioelectrical impedance (BIA), and dual energy X-ray absortiometry (DXA). BOD POD^® ^measurements were also performed in the Zaragoza sample.

Pubertal development was assessed according to the five established Tanner stages [47]. Each stage describes breast and pubic hair development in girls and genital and pubic hair development in boys.

Blood pressure was measured using a validated digital automatic blood pressure monitor (OMRON M6, OMRON HEALTH CARE Co., Ltd., Kyoto, Japan) according to the International Protocol of the European Society of Hypertension [48].

#### Haematological, biochemical, metabolic, immunological and genetic profile

Blood collection was performed upon an empty stomach between 8 and 10 AM, after fasting for 10 h. Health state of human volunteers was optimal for blood sample collection.

In all cases, blood was extracted from the antecubital vein (21.5 mL). Blood collection was carried out by experienced clinical staff. Blood samples were divided into aliquots as follows: 1.5 mL in EDTA tube (for haematological study and immunophenotyping of peripheral blood cells), 10 mL in EDTA tube (for plasma extraction) and 10 mL in gel containing tube (for serum extraction). To avoid erroneous values due to sample deterioration, blood cell counts and differentials were analyzed in the laboratories of each hospital that participated in this study. Within 1-h of collection, blood was centrifuged and aliquots of plasma or serum were stored at -80°C. Serum samples were sent to each laboratory at convenient time intervals (Madrid for the immunological study, Granada for the biochemical, metabolic and vitamin studies and Pamplona for the genetic study). Haematological, lipidic-metabolic, vitamins, immunological and genetic studies were centralized in each participating laboratory. The parameters included in each of these categories are presented in Tables 2, 3 and 4. Quality control of the assays was assured by the Regional Health Authority, fulfilling regulations for clinical hospital laboratories in Spain.

**Table 2:** Blood biochemical and metabolic profiles.

Biochemical variables	Analytical Techniques
Triglycerides, Total cholesterol, HDL-Cholesterol, HDL2/HDL3 fraction, LDL-cholesterol	Colorimetric assay
Apolipoprotein B, A1, Lipoprotein(a), ASP (acylation-stimulating protein)	Nephelometry, enzyme immuno assay (EIA) and radioimmuno assasy (RIA)
Non Esterified Free Acids (NEFA)	Chromatography

**Metabolical variables**	**Analytical Techniques**

Insulin, Glucose, Prealbumin, Uric acid, Homocysteine	Colorimetric assays, precipitation methods and nephelometry, enzyme immuno assay (EIA) and radioimmuno assasy (RIA)

**Oxidative state**	**Analytical Techniques**

Oxidised-LDL	Fluorimetry
Malonyl-dialdehyde	HPLC
Plasma total oxidative capacity	Spin electron resonance spectroscopy

**Vitamins**	**Analytical Techniques**

Tocopherol, retinol, vitamin C and folic acid	Chromatography

**Hormones**	**Analytical Techniques**

Thyrotropin (TSH), Grown hormone (GH), Follicle-stimulating hormone (FSH), Luteinizing hormone (LH), Adrenocorticotropin hormone (ACTH), Prolactin (PRL)	Human Pituitary Kit
Ghrelin, Insulin, Leptin, PYY	Gut Hormone Kit
Free tyroxine (FT4)	Radioimmuno assasy (RIA)

**Table 3:** Haematological and Immunological profiles.

Haematological profile	Analytical Techniques
Red blood cell counts and indices White blood cell counts and differential	Automatic cell counter (Beckman coulter)

**Cell-mediated immunity**	**Analytical Techniques**

Lymphocyte subsets:CD3, CD4, CD8, CD16/56, CD45RA, CD45RO	Flow Cytometry [55]

**Cellular immune function: Cytokines in serum**	**Analytical Techniques**

IL-1β, IL-2, IL-4, IL-5, IL-6, IL-8, IL-10, TNF-α	Immunoassay (x Map technology Linco kits) (56). Human High Sensitivity Kit.
TGF-β	Enzyme Linked Immunoabsorbent Assay (ELISA)

**Humoral immunity**	**Analytical Techniques**

Lymphocyte subset CD19	Flow Cytometry [55]
Humoral function: IgG, IgA, IgM, IgE	Nephelometry

**Innate immunity**	**Analytical Techniques**

C3, C4 and C-reactive protein.	Nephelometry

**Adhesion molecules**	**Analytical Techniques**

L-selectin, ICAM-1, VCAM-1	Immunoassay (x Map technology Linco kits) Cardiovascular CVD1 Kit
E-selectin	Enzyme Linked Immunoabsorbent Assay (ELISA)

**Adipokines**	**Analytical Techniques**

Adiponectin, leptin	Immunoassay (x Map technology Linco kits) Cardiovascular CVD1 Kit

**Table 4:** Genetic profile.

Genetic variables	Analytical Techniques
Adiponectin gene: A/C SNP -4034 (rs822395), T/G SNP +45 in exon 2 (rs2241766) G/T SNP +276 in intron 2 (rs1501299) [56] Interleukin 6 gene: 174 G/C SNP(rs1800795) [57] Fat mass associated gene (FTO): rs9939609 and rs7204609 [58]	ABI prism 7000 sequence detector followed by allelic discrimination
Melanocortin 4 receptor gene (MC4R) [59]	Sequencing of the entire coding region of the MC4R gene was performed on an automated DNA sequencer.

48 h urine samples were collected coinciding with the two last days of 72 h dietary records. Subjects were given detailed verbal and written instructions about how to collect a complete 48 h urine sample and given 3 L sterile plastic bottles. Collection began with the second urine of the second day and ended with the first urine of the last day of 72 h dietary record. Urine creatinine concentration was determined by kinetic Jaffe reaction on a Cobas centrifugal analyser (Roche, Montclair, NJ). Urinary Ca was determined by atomic absorption spectrometry (Atomic absorption spectrometer 1100B, Perkin Elmer, Norwalk, CT, USA). Phosphorous was analysed by photocolorimetry (Spectrophotometer PU8620 UV/VIS/NIR, Philips, Scientific and Analitycal Equipment, Eindhoven, The Netherlands). Quantitative urine control (Lyphochek^®^, Bio Rad Diagnostics Group, Irvine, CA, USA) was used to assess precision. Na^+^, Cl^- ^and K^+ ^were measured with an electrolyte analyser (EMLTM 100 Electrolyte Laboratory, Radiometer Copenhagen, Radiometer Medical A/S, Brønshøj, Denmark). Urine samples were diluted 2:1 (urine: diluent) with diluent for urine S2490 (Radiometer Copenhagen, Radiometer Medical A/S, Brønshøj, Denmark). QualitycheckTM S2480 and S2470 were used as internal standards (Radiometer, Copenhagen, Radiometer Medical A/S, Brønshøj, Denmark) to assess precision. Urine total volume and pH were also monitored.

### Ethical aspects

This project followed the ethical standards recognized by the Declaration of Helsinki (reviewed in Hong-Kong in September 1989 and in Edinburgh, Scotland in 2000) and the EEC Good Clinical Practice recommendations (document 111/3976/88, July 1990), and current Spanish legislation regulating clinical research in humans (Royal Decree 561/1993 on clinical trials). The study was approved by the Ethics Committee of each hospital that participated in this project and by the Ethics Committee of the Spanish Council for Scientific Research (CSIC). The study was explained to the participants before starting, and the volunteers, parents or tutors signed an informed consent.

### Data confidentiality

Access to the database was restricted to the researchers that participated in this study. Therefore, the information obtained in the study was considered as confidential, although the sanitary authorities have full access rights for inspection purposes.

### Statistical Analysis

The studied parameters are treated considering some fixed variability factors: gender, age, tanner, BMI classification [49]. Attendance, parent's presence and events are considered as random variability factors. Quality control includes a double data entry procedure in the data base.

Firstly, an assessment of missing data and the identification of potential outliers are carried out. The statistic processes is as follows:

1) Univariate descriptive analysis, study of data distribution, basic statistics such as central and dispersion values. The interrelationship among variables is assessed by studying the correlation coefficients on the basis of their distribution and their association to those groups defined upon random and fixed factors. Pair comparison tests with previous analysis of the homogeneity of variance are used. Chi-Squared tests and exact probability calculations are also performed to study the relationship among qualitative variables.

2) General lineal models for each point in time and as a function of time (longitudinal data analysis). Multivariate models are used from different perspectives: classical regression models and continuous or categorical principal component analysis are used to describe multivariate interrelationship among selected variables. Also, multivariate analyses are used to predict the intervention success at a selected period of time and at the end of the study by variables that measure the health improvement of the patients.

The analysis of the data will be done using the statistical packages SPSS and SAS.

## Discussion

The EVASYON study develops, for the first time in Spain, a multidisciplinary treatment programme for adolescents with overweight and obesity that is aimed at all possibly involved areas of the individual, such as dietary habits, physical activity and cognitive and psychological profiles, in order to prevent the development, in an immediate future and in the long term, of chronic diseases associated with obesity such as diabetes, hypertension, cardiovascular diseases, metabolic syndrome, etc. Health-related researchers who participate in this study expect that the programme, once its efficacy has been proved, may be applicable in any hospital or clinic from the Spanish System of Health with a multidisciplinary group consisting of paediatricians, endocrinologists, psychiatrists, psychologists, physical activity specialists and dieticians.

The EVASYON study is essentially characterized for being a multidisciplinary and multicentre project. Thus, both the assessment of the efficacy and the protocols of the treatment have been developed by professionals in each of the specific fields: psychology, nutrition, physical activity, fitness, paediatrics, body composition, genetics, immunology and biochemistry. Moreover, the study has been conducted in 5 hospitals from 5 Spanish cities, respectively (Madrid, Santander, Zaragoza, Pamplona and Granada). Adaptation of the protocol for 5 different hospitals had as principal advantage that the treatment programme potentially will be easier to implement in any healthcare centre of the Spanish Health Care Service.

Nonetheless, organizing a single educational treatment for all the centres and the assessment protocols was a complex process. Training workshops for all the EVASYON members, who were going to take part in the programme and in the assessment, were conducted to unify criteria and to test the methodologies presented in this article before beginning the study. One pilot group in each hospital served to solve starting-up problems in the treatment programme, doubts, human and technical resources and coordination with adolescents and their families. Some decisions were also made in the workshops on how to send blood samples, protocols of physical fitness test to carry out in hospitals, possible postbariatric surgery or follow-up of participants after the study ended.

The EVASYON study has both strengths and weaknesses. Several strengths that the EVASYON study presents are as follows: 1) the sample size achieved of 204 adolescents with overweight and obesity provides an acceptable statistical power to conduct multivariable analysis (e.g. sex, maturation status, compliance or not compliance of the educational therapeutic programme). Few studies performed in clinical settings have recruited, to our knowledge, a greater number of participants than the EVASYON study [24-26] the duration of the therapeutic programme is a one-year follow-up, which will make possible to observe long-term changes resulting from the intervention. In addition, it may reveal the differences that result between the intensive stage of the treatment and the extensive stage as reported in previous studies [50]; 3) The EVASYON study includes the most complete and comprehensive assessments of the effectiveness of a weight loss programme in the individual's physical and psychological health status. The 9 assessment categories represent a multitude of variables that will be evaluated throughout the study for being able to determine the most effective aspects of the intervention, and in which dimensions the effect of the intervention was the strongest one.

The EVASYON study also involves weaknesses that must be taken into account: 1) the study does not include a control group of overweight or obese adolescents. This makes it difficult to understand the progress, maintenance or deterioration of the baseline health of participants. Strong weight loss studies with educational therapeutic programme based on physical activity and nutrition have included control groups [51] or not [52]; 2) an important dropout due to the duration of the study was detected in participants and their families. Studies such as the one by Savoye et al. [51] include 209 subjects who were initially, recruited; 60% of the whole sample completed 6 months of treatment and only 53% completed 1-yr follow-up, obtaining therefore 47% loss of the adolescent participants during the study; 3) together with the loss of participants during the follow-up time, it also caused a loss of assessments. Participants and their families claimed the following reasons: forgetfulness, exam periods, inability to move a family member together with the adolescent, discouragement.

Initiatives as the EVASYON study contribute to the development of the Spanish Strategy for Nutrition, Physical Activity and the Prevention of Obesity (NAOS Strategy) initiated in 2005 by the Ministry of Health and Consumer Affairs [53]. The NAOS Strategy has as main aims to improve healthy eating and to increase physical activity levels. Therefore, informative campaigns, agreements with public and private institutions, voluntary working agreements, educational programmes and supporting health promotion initiatives are some of the activities carried out as part of the NAOS Strategy to invert the rate of obesity in Spanish population with special focus on children. The NAOS Strategy is in the line of the Global Strategy on Diet, Physical Activity and Health approved by the World Health Organization (WHO) in 2004 [54].

In summary, the EVASYON study is an interventional study assessing the effectiveness of an educational therapeutic model in physical activity and nutrition in Spanish adolescents with overweight and obesity. The EVASYON programme attempts to be a national pilot study that may be implemented as a method of treatment of obesity in adolescents into the Spanish Health Care Service. This multidisciplinary and multicenter study assesses changes in participants for approximately 1-year follow-up over 8 dimensions: 1) diet, 2) physical activity and health-related physical fitness, 3) psychology, 4) body composition, 5) haematology, 6) biochemical and metabolic profiles, 7) mineral and vitamin profiles, and 8) immunology profile. Genetic profile was also assessed for examining the influence of gene-environmental interactions on obesity.

## Competing interests

The authors declare that they have no competing interests.

## Authors' contributions

DMG, SGM, and MAP contributed equally to this work. AM, CRF, CC, AM, JMG designed the study and obtained funding. DMG, SGM, MAP, EN, JW, OLV, CA, MPV, MD, JAM, MGF and LAM provided insight into the study design. All authors participated in the writing of the paper and provided comments on the drafts and approved the final version.

## Pre-publication history

The pre-publication history for this paper can be accessed here:

http://www.biomedcentral.com/1471-2458/9/414/prepub
